# Dissecting photosynthetic electron transport and photosystems performance in Jerusalem artichoke (*Helianthus tuberosus* L.) under salt stress

**DOI:** 10.3389/fpls.2022.905100

**Published:** 2022-07-27

**Authors:** Kun Yan, Huimin Mei, Xiaoyan Dong, Shiwei Zhou, Jinxin Cui, Yanhong Sun

**Affiliations:** ^1^School of Agriculture, Ludong University, Yantai, China; ^2^School of Life Sciences, Liaoning University, Shenyang, China; ^3^CAS Key Laboratory of Coastal Environmental Processes and Ecological Remediation, Yantai Institute of Coastal Zone Research, Chinese Academy of Sciences (CAS), Yantai, China; ^4^School of Environmental and Material Engineering, Yantai University, Yantai, China

**Keywords:** chlorophyll fluorescence, delayed chlorophyll fluorescence, malondialdehyde, modulated 820 nm reflection, photoinhibition

## Abstract

Jerusalem artichoke (*Helianthus tuberosus* L.), a vegetable with medical applications, has a strong adaptability to marginal barren land, but the suitability as planting material in saline land remains to be evaluated. This study was envisaged to examine salt tolerance in Jerusalem artichoke from the angle of photosynthetic apparatus stability by dissecting the photosynthetic electron transport process. Potted plants were exposed to salt stress by watering with a nutrient solution supplemented with NaCl. Photosystem I (PSI) and photosystem II (PSII) photoinhibition appeared under salt stress, according to the significant decrease in the maximal photochemical efficiency of PSI (△MR/MR_0_) and PSII. Consistently, leaf hydrogen peroxide (H_2_O_2_) concentration and lipid peroxidation were remarkably elevated after 8 days of salt stress, confirming salt-induced oxidative stress. Besides photoinhibition of the PSII reaction center, the PSII donor side was also impaired under salt stress, as a K step emerged in the prompt chlorophyll transient, but the PSII acceptor side was more vulnerable, considering the decreased probability of an electron movement beyond the primary quinone (ETo/TRo) upon depressed upstream electron donation. The declined performance of entire PSII components inhibited electron inflow to PSI, but severe PSI photoinhibition was not averted. Notably, PSI photoinhibition elevated the excitation pressure of PSII (1-qP) by inhibiting the PSII acceptor side due to the negative and positive correlation of △MR/MR_0_ with 1-qP and ETo/TRo, respectively. Furthermore, excessive reduction of PSII acceptors side due to PSI photoinhibition was simulated by applying a specific inhibitor blocking electron transport beyond primary quinone, demonstrating that PSII photoinhibition was actually accelerated by PSI photoinhibition under salt stress. In conclusion, PSII and PSI vulnerabilities were proven in Jerusalem artichoke under salt stress, and PSII inactivation, which was a passive consequence of PSI photoinhibition, hardly helped protect PSI. As a salt-sensitive species, Jerusalem artichoke was recommended to be planted in non-saline marginal land or mild saline land with soil desalination measures.

## Introduction

As a major abiotic stress endangering agricultural production, soil salinity usually lies in farmland with irrational irrigation and saline land in inland arid and coastal regions (Nikalje et al., 2017). Under salt stress, plants are first confronted with osmotic stress and then have to endure ionic toxicity; however, the damages to biological macromolecules often resulted from the salt-induced excess generation of reactive oxygen species (ROS) in plant cells (Gill and Tuteja, 2010; Hossain and Dietz, 2016; Chen et al., 2018). In photosynthetic organisms, ROS can be considered a by-product of photosynthetic electron transport in the chloroplast (Asada, 2006; Gill and Tuteja, 2010; Foyer, 2018).

Photosynthetic electron transport from water to NADP^+^ is powered by photosystem II (PSII) and photosystem I (PSI), and this electron transport chain also involves other electron carriers such as oxygen-evolving complex, primary and secondary quinone (Q_A_ and Q_B_), and plastoquinone (PQ). In contrast to the equilibrium state of ROS in plants under normal growth condition, depressed CO_2_ assimilation will inhibit photosynthetic electron transport in a feedback way and then elevate excitation pressure in the chloroplast under abiotic stress (Murata et al., 2007; Takahashi and Murata, 2008; Zhang et al., 2014; Yan et al., 2015, 2018b). As a consequence, a great number of photosynthetic electrons tend to be transferred to O_2_ rather than NADP^+^ to generate superoxide anion (O_2_^–^). Hydrogen peroxide (H_2_O_2_) is generated from O_2_^–^ through dismutation reaction, and then hydroxyl radical, the most dangerous ROS, may be synthesized by the Fenton reaction finally (Gill and Tuteja, 2010; Foyer, 2018). In addition, the elevated excitation pressure can also cause greater production of singlet oxygen in PSII reaction centers (Foyer, 2018). The excess generation of these ROS may bring about PSII and PSI photoinhibition by impairing photosynthetic membrane proteins or lipids. In particular, Oukarroum et al. (2015) illustrated that PSI and PSII photochemical capacities were negatively correlated with ROS production. At present, salt-induced PSII photoinhibition has been extensively documented. Traditionally, PSII is considered more vulnerable than PSI under abiotic stresses, and rapid PSII photoinhibition can protect PSI by reducing ROS production at its acceptor side by restricting electron flow to PSI under light stress or high temperature (Yan et al., 2013a,b; Zivcak et al., 2014; Zhang et al., 2016). In contrast, limited restriction on PSII electron donation is liable to induce PSI photoinhibition under chilling stress (Zhang et al., 2011, 2014; Yang et al., 2014). PSI was also demonstrated to be a possible photoinhibition site under salt stress in our recent study, as PSII photoinhibition hardly prevented PSI photoinhibition in a salt-sensitive honeysuckle cultivar (Yan et al., 2015). Compared with PSII photoinhibition, PSI photoinhibition is more harmful in light of its difficult recovery (Sonoike, 2011), and particularly, PSI vulnerability poses a big threat to PSII by aggravating feedback inhibition at the PSII acceptor side. Therefore, PSII and PSI interaction is very important for plants to adapt to abiotic stress. To date, less attention has been paid to the salt tolerance of PSI than PSII, and moreover, PSII and PSI interaction remain largely unknown under salt stress. In other words, the characterization of photosynthetic electron transport has not been thoroughly dissected in plants under salt-induced oxidative stress, since PSII and PSI interaction relies on this electron transport process.

The Jerusalem artichoke (*Helianthus tuberosus* L.) is a vegetable native to North America. The Jerusalem artichoke can be used for medical applications and ethanol production because the tubers contain quantities of fructose and inulin (Baldini et al., 2004; Saengthongpinit and Saijaanantakul, 2005). In recent years, we have analyzed photosynthetic characteristics at various leaf expansion stages, verified PSII susceptibility to high temperature, and particularly demonstrated the sensitivity to waterlogging from aspects of PSI vulnerability and photosynthesis in Jerusalem artichoke (Yan et al., 2012, 2013b, 2018b). Notably, Jerusalem artichoke has been selected for an attempt to utilize marginal land in the coastal zone in China, considering its high capacity to acclimate to barren soil (Long et al., 2016). It has been reported that salt stress can decrease CO_2_ assimilation and induce oxidative injury with chlorophyll loss in Jerusalem artichoke (Long et al., 2009; Huang et al., 2012; Li et al., 2017). However, the stability of photosystems has not been paid enough attention in Jerusalem artichoke under salt-induced oxidative stress, let alone the characterization of photosynthetic electron transport.

A new technique has been recently developed to simultaneously detect prompt chlorophyll fluorescence (PF), modulated 820 nm reflection (MR), and delayed chlorophyll fluorescence (DF) (Goltsev et al., 2009; Strasser et al., 2010; Gao et al., 2014; Yan et al., 2018a). In this study, we aimed to investigate photosystems performance and interaction by analyzing the photosynthetic electron transport process in Jerusalem artichoke under salt-induced oxidative stress using this technique. This study can deeply unveil the mechanism of plant resistance to salt stress and may aid in the exploitation of marginal abandoned land.

## Materials and methods

### Plant material and treatment

In Laizhou Bay, China, Jerusalem artichoke tubers were gathered and cultivated in the room as in the previous study (Yan et al., 2018b). The tubers were planted in plastic pots (20 cm in diameter and 25 cm high) filled with vermiculite and cultured in an artificial climatic room (Qiushi, China). There was one tuber in each pot, and the vermiculite was kept wet by watering. The photon flux density, day/night temperature, and humidity were controlled at 400 μmol m^–2^ s^–1^ (12 h/day from 07:00 to 19:00), 25/18°C, and 70% in the room, respectively. After 1 month, the germinated seedlings appeared, and their growth was ensured by daily watering with Hoagland nutrient solution (pH 5.7). After 1 month, 45 uniform seedling plants were chosen and divided into three groups. In the first group, the control plants were not subjected to NaCl stress. In the second group, plants were subjected to 100 mM NaCl stress. In the third group, plants were subjected to 200 mM NaCl stress. NaCl was added to the nutrient solution gradually by 50 mM step every day to the final treatment concentrations (100 and 200 mM) on the same day, and thereafter, the salt stress persisted for 8 days. During the salt treatment experiment, the solution was refreshed every 2 days, and before refreshing the solution, the culture substrate was thoroughly leached using the nutrient solution to avoid ion accumulation. The newest fully expanded leaves were sampled to measure physiological and biochemical parameters.

### Assay of Na^+^, H_2_O_2_, malondialdehyde, and relative water contents

Fresh leaf tissues were sampled for measuring MDA, H_2_O_2_, Na^+^, and relative water contents using colorimetric methods, and the detailed procedure was reported in our previous studies (Yan et al., 2015, 2018b).

### Test of gas exchange with modulated chlorophyll fluorescence

An open photosynthetic system (LI-6400XTR, Li-Cor, Lincoln, NE, United States) equipped with a fluorescence leaf chamber (6400-40 LCF, Li-Cor) was utilized, and the same measuring procedure in our previous study was adopted for measuring the photosynthetic rate (Pn) and stomatal conductance (g_s_) (Yan et al., 2018b). The actual photochemical efficiency of PSII (ΦPSII) and photochemical quenching coefficient were also recorded, and then PSII excitation pressure (1-qP) was calculated.

### Detection of prompt chlorophyll fluorescence, modulated 820 nm reflection transients, and delayed chlorophyll fluorescence

The detection of PF, DF, and MR transients was simultaneously conducted using a multifunctional plant efficiency analyzer (MPEA, Hansatech, Norfolk, United Kingdom) with the same illumination procedure as in our previous study (Yan et al., 2018a). According to Schansker et al. (2003) and Strasser et al. (2010), the maximal photochemical efficiencies of PSII (Fv/Fm) and PSI (ΔMR/MR_0_), Q_A_ reducing reaction centers per PSII antenna chlorophyll (RC/ABS), variable fluorescence intensity at K step (V_k_), the probability with which an electron moves beyond Q_A_ (ETo/TRo), and from the intersystem electron carriers to reduce PSI end electron acceptors (REo/ETo) were calculated.

### Statistical analysis

One-way ANOVA was performed using SPSS 16.0 (SPSS Inc., Chicago, IL, United States) for all data, which are the average value from five replicate plants. The average value was compared through the LSD test. Regression analysis of △MR/MR_0_ with 1-qP and ETo/TRo was also performed using SPSS 16.0.

## Results

### Lipid peroxidation, H_2_O_2_, Na^+^, and relative water contents

**FIGURE 1:**
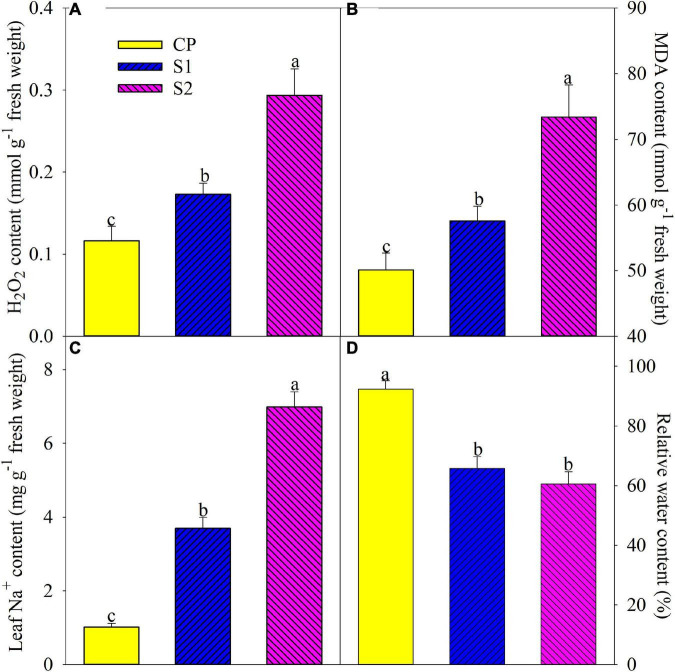
Changes in leaf H_2_O_2_ **(A)**, malondialdehyde (MDA) **(B)**, Na^+^ **(C)**, and relative water **(D)** contents in Jerusalem artichoke after 8 days of 100 and 200 mM NaCl stress. Data in the figure are the average value of five replicates (±SD), and the different letters on error bars indicate remarkable differences among salt treatments at *P* < 0.05. CP, T1, and T2 indicate control plants, plants exposed to 100 and 200 mM NaCl, respectively, and these symbols are also used in the following figures.

The level of lipid peroxidation in plant tissues can be reflected by MDA content. After 8 days of 100 mM NaCl stress, H_2_O_2_, and MDA contents were significantly elevated by 48.89 and 14.86% in the leaves of Jerusalem artichoke, and the increase was up to 152.46 and 46.42% under 200 mM NaCl stress (Figures 1A,B). Leaf Na^+^ was significantly increased by 2.65– and 5.92-fold after 8 days of 100 and 200 mM NaCl stress, respectively (Figure 1C). Leaf relative water content remarkably decreased on day 8 under 100 and 200 mM NaCl stress, and there was no significant difference in leaf relative water content between the two salt treatments (Figure 1D).

### Photosynthetic rate, stomatal conductance, photosystem II actual quantum yield, and excitation pressure

**FIGURE 2:**
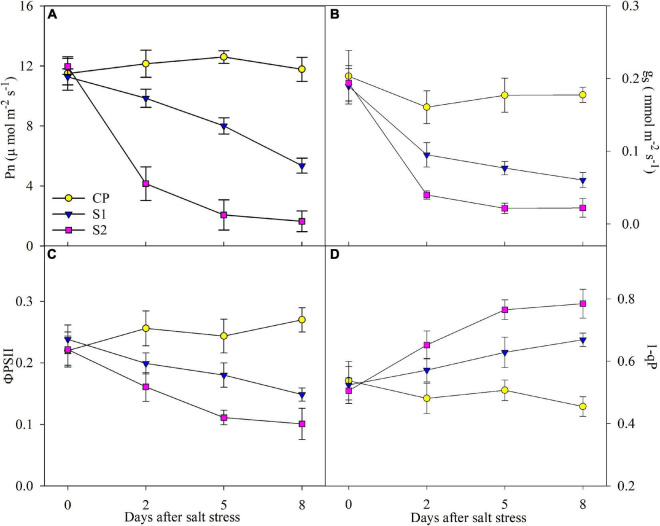
Changes in photosynthetic rate (Pn) **(A)**, stomatal conductance (g_s_) **(B)**, actual photochemical efficiency of photosystem II (PSII) (ΦPSII) **(C)**, and PSII excitation pressure (1-qP) **(D)** in Jerusalem artichoke under 100 and 200 mM NaCl stress. Data in the figure are the average value of five replicates (±SD), and the different letters on error bars indicate remarkable differences among salt treatments at *P* < 0.05.

After 2 days of 100 mM NaCl stress, Pn, g_s_, and ΦPSII were significantly reduced, and the reduction was up to 52.51, 68.09, and 37.66% on day 8 (Figures 2A–C). In comparison, the reduction of Pn, g_s_, and ΦPSII was far greater under 200 mM NaCl stress (Figures 2A–C). Under 100 mM NaCl stress, 1-qP was significantly elevated on day 2, and the elevation reached 27.67% on day 8, whereas the elevation of 1-qP was greater under 200 mM NaCl stress (Figure 2D).

### Prompt chlorophyll fluorescence, modulated 820 nm reflection transients, and delayed chlorophyll fluorescence

**FIGURE 3:**
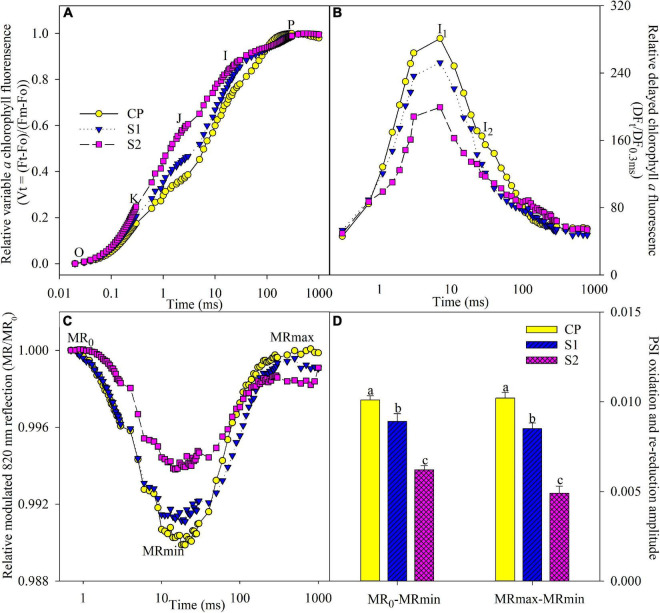
Transients of prompt chlorophyll fluorescence **(A)**, delayed chlorophyll fluorescence **(B)**, modulated 820 nm reflection **(C)**, and photosystem I (PSI) oxidation and re-reduction amplitude **(D)** in Jerusalem artichoke after 8 days of 100 and 200 mM NaCl stress. The specific steps in chlorophyll fluorescence transient are O, K, J, I, and P. The value of modulated 820 nm at the onset of red light illumination [0.7 ms, the first reliable modulated reflection (MR) measurement] is MR_0_. PSI oxidation and re-reduction amplitude were represented by MR_0_–MR_min_ and MR_max_–MR_min_, respectively. Data of MR_0_–MR_min_ and MR_max_–MR_min_ indicate the average value of five replicates (±SD), and the different letters on error bars indicate significant differences at *P* < 0.05. In delayed chlorophyll fluorescence curves, D0, I1, I2, and D2 are the initial point, the first (7 ms) and second (50 ms) maximal peaks, and the minimum point. The initial microsecond delayed fluorescence signal at 0.3 ms is indicated by DF_0_._3 *ms*_. The signals were plotted on a logarithmic timescale, and each curve is the mean of five replicate plants.

J and I steps indicate the accumulation of reduced Q_A_ and PQ (Schansker et al., 2003, 2005; Yan et al., 2013b). J and I steps obviously rose under salt stress on day 8, suggesting that PQ and Q_A_ re-oxidation were inhibited (Figure 3A). The occurrence of K step around 300 μs suggests the injury on OEC at the PSII donor side (Oukarroum et al., 2013, 2016). After 8 days of 200 mM NaCl stress, the PSII donor side was damaged, as indicated by the occurrence of the K step (Figure 3A). In contrast, J and I steps were less elevated, and the K step did not appear under 100 mM NaCl stress (Figure 3A).

During PSI oxidation, MR_0_ decreased to the minimal value (MR_min_). Subsequently, PSI re-reduction was initiated, and MR/MR_0_ increased to the maximal level (MR_*max*_). MR transient was remarkably changed by salt stress, as MR_0_–MR_min_ and MR_max_–MR_min_ significantly decreased (Figures 3C,D), suggesting the negative effect on both PSI oxidation and re-reduction, and the variations were greater in plants under 200 mM NaCl stress than 100 mM NaCl stress (Figures 3C,D). Under salt stress, DF transient was prominently suppressed in line with lowered I_1_ and I_2_ peaks, and obviously, the influence was less in plants under 100 mM NaCl stress than 200 mM NaCl stress (Figure 3B).

### Photosynthetic electron transport process

**FIGURE 4:**
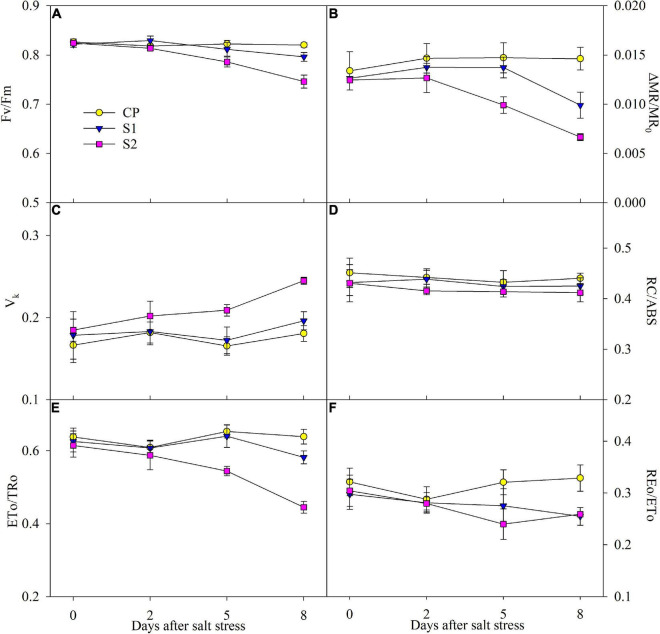
Changes in the maximal photochemical efficiency of photosystem II (PSII) (Fv/Fm) **(A)**, photosystem I (PSI) (△MR/MR_0_) **(B)**, variable fluorescence intensity at K step (V_k_) **(C)**, primary quinone reducing reaction centers per PSII antenna chlorophyll (RC/ABS) **(D)**, probability that an electron moves beyond primary quinone (ETo/TRo) **(E)**, and probability with which an electron from the intersystem electron carriers is transferred to reduce end electron acceptors at the PSI acceptor side (REo/ETo) **(F)** in Jerusalem artichoke under 100 and 200 mM NaCl stress. Data in the figure are the average value of five replicates (±SD), and the different letters on error bars indicate remarkable differences among salt treatments at *P* < 0.05.

After 200 mM NaCl stress for 5 days, △MR/MR_0_ and Fv/Fm were significantly reduced, and the reduction was up to 54.31 and 9.06% on day 8 (Figures 4A,B). After 8 days of 100 mM NaCl stress, the obvious decrease of 32.15 and 2.94% appeared in △MR/MR_0_ and Fv/Fm, respectively (Figures 4A,B). The greater decrease in △MR/MR_0_ than Fv/Fm implied that PSI photoinhibition was more severe than PSII photoinhibition under NaCl stress. After 5 days of 200 mM NaCl stress, ETo/Tro, and REo/ETo significantly declined, while the marked decrease in them was not found until 8 days of 100 mM NaCl stress (Figures 4E,F). No obvious effect on V_k_ was noted under 100 mM NaCl stress, but it was significantly increased after 200 mM NaCl stress for 5 days (Figure 4C). Under salt stress, only a mild decrease was observed in RC/ABS (Figure 4D).

### The coordination between photosystem I and photosystem II

**FIGURE 5:**
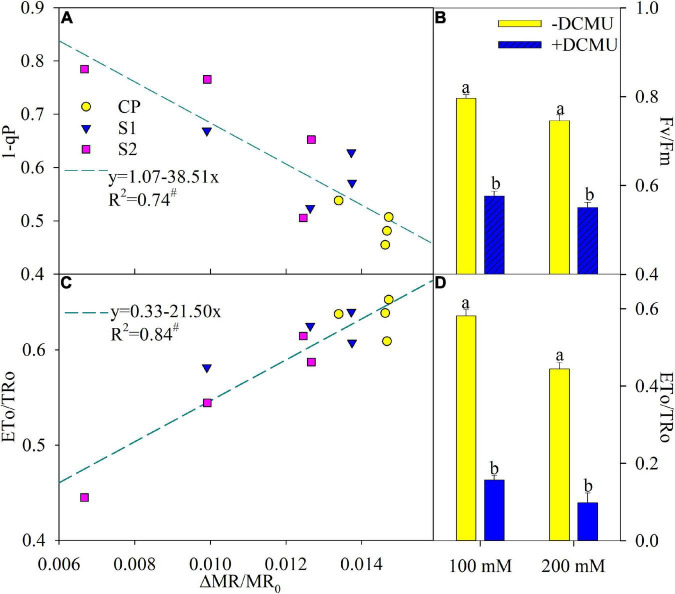
Regression of the maximal photochemical efficiency of photosystem II (PSI) (△MR/MR_0_) with PSII excitation pressure (1-qP) **(A)** and probability that an electron moves beyond primary quinone (ETo/TRo) **(C)** in Jerusalem artichoke. The significant correlation at *P* < 0.05 was indicated by #. Effects of applying DCMU on the maximal photochemical efficiency of PSII (Fv/Fm) **(B)** and ETo/TRo **(D)** in Jerusalem artichoke after 8 days of 100 and 200 mM NaCl stress. For reagent treatment, the leaves after 5 days of 100 and 200 mM NaCl stress were immersed in 0 or 70 μM DCMU for 3 h in the dark. Data of Fv/Fm and ETo/TRo indicate the average value of five replicates (±SD), and the different letters on error bars indicate remarkable differences between the leaves with and without DCMU treatment at *P* < 0.05.

According to the regression analysis, △MR/MR_0_ had a significant positive correlation with ETo/TRo, whereas, the correlation between 1-qP and △MR/MR_0_ was markedly negative (Figures 5A,C). DCMU functioned as a specific inhibitor for intervening electron transport from Q_A_^–^ to Q_B_^–^, and Fv/Fm and ETo/TRo were significantly decreased in plants applied with DCMU than those without DCMU application after 8 days of salt stress (Figures 5B,D).

## Discussion

As an ordinary finding, photosynthesis was depressed by salt stress in line with stomatal closure in Jerusalem artichoke (Figures 2A,B). The inhibited CO_2_ fixation can cause feedback inhibition on photosynthetic electron transport and accelerate ROS production as more photosynthetic electrons are diverged to oxygen (Gill and Tuteja, 2010; Foyer, 2018). Exactly, the elevated leaf lipid peroxidation and H_2_O_2_ concentration proved salt-induced oxidative stress on Jerusalem artichoke (Figures 1A,B). Elevated ROS generation in photosynthetic organisms is usually associated with the inhibited photosynthetic electron transport and can cause photosystems photoinibition with oxidative damage to photosynthetic membranes lipids and proteins (Murata et al., 2007; Sonoike, 2011; Oukarroum et al., 2015). Therefore, photosystem photoinhibition seems to be a feasible proxy for the oxidative threat to the plant (Zhang et al., 2012, 2014; Yan et al., 2015, 2018b). Under salt stress, Na^+^ toxicity may induce more severe oxidative stress on photosystems than osmotic pressure (Muranaka et al., 2002; Allakhverdiev and Murata, 2008; Cha-um and Kirdmanee, 2010; Hossain et al., 2017). In this study, leaf oxidative damage also resulted from Na^+^ toxicity rather than osmotic pressure to a greater extent, as severe lipid peroxidation appeared with greater leaf Na^+^ accumulation rather than leaf water deficit under salt stress with 200 mM NaCl than 100 mM NaCl (Figures 1C,D).

Consistent with leaf ROS burst, salt stress actually caused PSI and PSII photoinhibition according to the significantly lowered Fv/Fm and △MR/MR_0_ in Jerusalem artichoke (Figures 4A,B). The classic proxy for the photochemical capability of the PSII reaction center, Fv/Fm rarely reflects PSII whole performance (Li et al., 2009). Under 200 mM NaCl stress, the elevated J step and declined ETo/Tro suggested the inhibited electron transport beyond Q_A_ with accumulated Q_A_^–^, while electron donation from the oxygen-evolving complex was also constrained due to the increased V_k_ (Figures 3A, 4C,E). I_1_ peak indicating the accumulation of S3Z^+^P680Q_A_^–^ can comprehensively reflect the state of the whole PSII, including active reaction centers and electron transporters at both donor and acceptor sides (Goltsev et al., 2009; Gao et al., 2014). Depressed I_1_ corroborated salt-induced damage on PSII (Figure 3B). The value of ETo/TRo is dependent not only on electrons transferred beyond Q_A_ but also on electrons donation from upstream electron carriers. Thus, the PSII acceptor side exhibited greater salt susceptibility than the reaction center and donor side in view of the significant reduction in ETo/TRo on the premise of lowered electron donation from the upstream under 200 mM NaCl stress. Consistently, the similar variations of ETo/TRo, Fv/Fm, and V_k_ under 100 mM NaCl stress also verified the greater susceptibility of the PSI acceptor side (Figures 4A,C,E). In addition, unchanged V_k_ and K step with lowered Fv/Fm under 100 mM NaCl stress suggested that salt sensitivity of the PSII donor side was lower than the PSII reaction center (Figures 4A,C). In summary, the salt sensitivity of PSII components gradually rose along with the direction of photosynthetic electron transport. The responses of whole PSII components also implied PSII vulnerability in Jerusalem artichoke under salt stress.

The declined PSII performance was consistent with the elevated PSII excitation pressure upon declined CO_2_ assimilation and restricted electron flow to PSI when photosynthesis reached a steady-state (Figures 2A,C,D). In MR transients, the lowered PSI re-reduction amplitude also suggested the restricted electron donation from PSII (Figures 3C,D). The restricted electron donation from PSII can help protect PSI against photoinhibition by decreasing the probability of ROS generation at the PSI acceptor side. However, PSI photoinhibition was never prevented under salt stress and was even more severe than PSII photoinhibition, considering the greater decreased amplitude of △MR/MR_0_ than Fv/Fm (Figures 4A,B). Limited electron inflow should improve PSI oxidation by blocking its re-reduction in MR transients; however, PSI oxidation was curtailed with decreased PSI oxidative amplitude, confirming that PSI encounters greater damage than PSII (Figures 3C,D). Because the reopening of PSII reaction centers is prolonged by electron transfer from reduced quinone to plastoquinone before the plastoquinone pool is fully reduced, an I_2_ phase appears in DF transient (Goltsev et al., 2009). Salt-induced decrease in I_2_ coincided with decreased REo/ETo and elevated I step, and all these changes pointed to that salt-induced PSI damage led to inhibition of PQ re-oxidation (Figures 3A,B, 4F). To summarize, PSI was more vulnerable to salt stress than PSII in Jerusalem artichoke, but in disagreement with the traditional viewpoint, PSII inactivation offered scarce protection to PSI.

In accordance with the negative correlation of △MR/MR_0_ with 1-qP and the positive correlation of △MR/MR_0_ with ETo/TRo (Figures 5A,C), PSI photoinhibition led to feedback inhibition on PSII electron outflow at the acceptor side and then elevated exciting pressure of PSII in Jerusalem artichoke upon salt stress. In addition, over-reduction of PSII acceptor side due to PSI photoinhibition was simulated by the experiment of DCMU application, and the result demonstrated that PSII photoinhibition was actually accelerated by PSI photoinhibition in Jerusalem artichoke under salt stress (Figures 5B,D). Thus, salt-induced depression on PSII performance should be interpreted as a result of PSI photoinhibition, and the passive PSII inactivation was rarely capable of defending PSI oxidative injury. Invalid PSII and PSI interaction has been found with PSI vulnerability in sensitive plants under abiotic stress and can bring about detrimental effects on the entire photosynthetic apparatus (Zhang et al., 2014; Yan et al., 2018b). Accordingly, Jerusalem artichoke should be classified as a salt-sensitive plant.

## Conclusion

Photosystem II and PSI vulnerability to salt stress were illustrated in Jerusalem artichoke, and PSII inactivation, which was a passive consequence of PSI photoinhibition, hardly helped defend PSI. Given the salt sensitivity of Jerusalem artichoke, it is better to select non-saline marginal land for planting in agricultural practice, or the mild saline land in the coastal zone can also be used in combination with some desalination measures such as freshwater leaching and applying salt separation layer.

## Data availability statement

The raw data supporting the conclusions of this article will be made available by the authors, without undue reservation.

## Author contributions

KY designed and performed the experiment and wrote the manuscript. HM, JC, and YS participated in the experiment. XD and SZ participated in the data analysis. All authors have read the manuscript and approved the final version of the manuscript.

## Conflict of interest

The authors declare that the research was conducted in the absence of any commercial or financial relationships that could be construed as a potential conflict of interest.

## Publisher’s note

All claims expressed in this article are solely those of the authors and do not necessarily represent those of their affiliated organizations, or those of the publisher, the editors and the reviewers. Any product that may be evaluated in this article, or claim that may be made by its manufacturer, is not guaranteed or endorsed by the publisher.

## Abbreviations

ETo/TRo: the probability for an electron movement beyond primary quinone
g_s_: stomatal conductance
MDA: malondialdehyde
Pn: photosynthetic rate
PSI: photosystem I
PSII: photosystem II
RC/ABS: primary quinone reducing reaction centers per PSII antenna chlorophyll
PQ: plastoquinone
REo/ETo: the probability with which an electron from the intersystem electron carriers is transferred to reduce end electron acceptors at the PSI acceptor side
Q_A_: primary quinone
ROS: reactive oxygen species
V_k_: variable fluorescence intensity at K step
△ MR/MR_0_: the maximal photochemical capacity of PSI
Φ PSII: actual photochemical efficiency of PSII
1-qP: excitation pressure of PSII.
